# Honey bee (*Apis mellifera*) nurses do not consume pollens based on their nutritional quality

**DOI:** 10.1371/journal.pone.0191050

**Published:** 2018-01-11

**Authors:** Vanessa Corby-Harris, Lucy Snyder, Charlotte Meador, Trace Ayotte

**Affiliations:** Carl Hayden Bee Research Center, USDA-ARS, Tucson, Arizona, United States of America; Philipps-Universitat Marburg Fachbereich Biologie, GERMANY

## Abstract

Honey bee workers (*Apis mellifera*) consume a variety of pollens to meet the majority of their requirements for protein and lipids. Recent work indicates that honey bees prefer diets that reflect the proper ratio of nutrients necessary for optimal survival and homeostasis. This idea relies on the precept that honey bees evaluate the nutritional composition of the foods provided to them. While this has been shown in bumble bees, the data for honey bees are mixed. Further, there is controversy as to whether foragers can evaluate the nutritional value of pollens, especially if they do not consume it. Here, we focused on nurse workers, who eat most of the pollen coming into the hive. We tested the hypothesis that nurses prefer diets with higher nutritional value. We first determined the nutritional profile, number of plant taxa (richness), and degree of hypopharyngeal gland (HG) growth conferred by three honey bee collected pollens. We then presented nurses with these same three pollens in paired choice assays and measured consumption. To further test whether nutrition influenced preference, we also presented bees with natural pollens supplemented with protein or lipids and liquid diets with protein and lipid ratios equal to the natural pollens. Different pollens conferred different degrees of HG growth, but despite these differences, nurse bees did not always prefer the most nutritious pollens. Adding protein and/or lipids to less desirable pollens minimally increased pollen attractiveness, and nurses did not exhibit a strong preference for any of the three liquid diets. We conclude that different pollens provide different nutritional benefits, but that nurses either cannot or do not assess pollen nutritional value. This implies that the nurses may not be able to communicate information about pollen quality to the foragers, who regulate the pollens coming into the hive.

## Introduction

Insect pollinators are vital to our ecosystem and food supply. Multiple interacting factors threaten pollinator species richness and population size, including disease, pesticides, climate change, and lack of adequate forage [1, 2]. While many of these factors are beyond our immediate control due to a variety of economic and geopolitical challenges, nutrition is something that we might be able to make greater progress on through informed landscape management decisions [3]. These decisions rely on basic information about the nutritional value of pollens for individuals and, in the case of social insect pollinators like honey bees, their colonies.

Honey bees consume pollen and nectar from flowers. Nectar provides carbohydrates and small amounts of other nutrients, while pollen provides the bulk of the insect’s protein and lipid requirements. Pollen is a key component of honey bee individual and colony health, impacting growth, longevity, and immunocompetence [4–24]. While foragers consume large amounts of nectar and honey to provide the energy for flight, nurse worker bees (“nurses”, ~5–15 days of age [25]) eat large amounts of pollen in order to fuel the growth and secretory activity of their hypopharyngeal glands (HGs). These paired secretory glands in the head secrete the major protein fraction of the jelly fed via trophallaxis to larvae, adult workers, and queens in the hive. HGs are sensitive to pollen intake, and a hallmark of nurse pollen deprivation is their small HGs, which actively degrade in response to pollen deprivation [13]. HG development is also sensitive to pollen source, with different pollens and pollen mixtures contributing to this variability [5, 6]. But despite detailed data on the plant taxon diversity and nutrient content of pollens that result in HG growth [4, 6], it is still unclear what components of these pollens encourage HG growth.

Many herbivorous insects prefer diets that best meet their nutritional needs and that provide a homeostatic benefit [26, 27]. Honey bees are polylectic, but when they are provided with a variety of plants to forage on they tend to concentrate their efforts on certain plant taxa [28–31]. One reason for this may be that these preferred plants provide a greater nutritional reward. However, in contrast to bumblebees [32–36], it is not entirely clear from the literature whether honey bees choose pollens according to its nutritional value [32, 37–42]. Multiple studies find that foragers do not prefer high- versus low-quality pollens [38, 43]. Pernal and Currie found that foragers do not recruit to pollens that rescue experimentally-imposed protein deficiencies, but instead rescue these deficiencies by foraging more overall [40]. Additionally, Leonhardt and Blüthgen [32] found that honey bees collect lower-quality pollens than bumblebees foraging in the same area, suggesting that honey bees do not have the same capacity as bumblebees to evaluate pollen quality. However, recent work suggests that honey bees do, in fact, choose diets based on their quality. For example, Hendriksma and Shafir [44] found that honey bee foragers that were fed for a week with flours deficient in certain amino acids preferred flour-based diets that complemented those deficiencies once these diets were provided. Zarchin *et al*. [45] later showed that foragers fed pollens deficient in certain fatty acids for 2–5 days preferred pollens that were then provided afterwards that complemented these deficiencies. Importantly, both studies showed that foragers could discriminate the diets based on odor, providing a mechanism for how foragers might choose these complementary diets. But in a colony, foragers do not consume and digest most of the pollen; nurses do [46, 47]. Nurses are also the most sensitive to hive pollen deficiencies and should be the first ones to communicate their pollen needs to foragers. Indeed, pollen-starved nurses do communicate these needs to foragers, presumably through touch or trophallaxis of non-proteinaceous substances [48–51]. This nurse-forager communication as well as the foragers’ direct assessment of pollen stores [52–55] and brood pheromone [56] can stimulate pollen foraging. But what happens when the quality, and not necessarily the quantity, of pollen is insufficient to meet brood-rearing activities in the hive? These complex and intimate signals between nurses, who consume and possibly evaluate the pollen, and foragers may be especially important for increasing foraging efficiency in environments containing both high- and low-quality pollens.

If nurses communicate their needs to foragers, the assumption is that nurse bees can first assess resource quality, determine if it meets their needs, and communicate to the foragers whether the diet is adequate or not. We were particularly interested in the first part of this assumption, that nurse bees assess pollen quality. In paired choice experiments, nurses optimize their intake of essential amino acids and carbohydrates in order to maximize their survival [57], suggesting that they can evaluate their diet and consume higher quality diets accordingly. However, the liquid diet provided in [57] was arguably less complex than pollen, which is made up of a thin exine layer surrounding a nutritious intine. We reasoned that, if nurses assessed the quality of pollen diets, choice experiments would indicate higher consumption of the more nutritious pollens compared to the less nutritious pollens. We therefore conducted a series of experiments in nurse-aged honey bees to explore the connection between pollen nutrition and choice. We first catalogued the nutritive properties and plant taxon richness for three different pollens collected by honey bees. We then tested whether nurse bees chose more nutritious pollens over others. We also conducted similar tests using supplemented pollen and liquid diets to further test whether nutrition plays a role in choice. Although the nutritional value of the three pollens differed, nutrition did not influence choice. This suggests that, although certain pollens provide a greater benefit per unit of consumption, honey bee nurses either cannot or do not discriminate among pollens based on nutrition.

## Materials and methods

### Source pollens

Three types of honey bee-collected pollens were used for the assays described below: “almond” pollen collected in California in February of 2014, “desert” pollen collected in Tucson in the spring of 2015, and “Southeast” (“SE”) pollen purchased from Durham’s Bee Farm (www.durhamsbeefarm.com) in the fall of 2015. All pollens were kept at -20°C while in the hands of the USDA-ARS Carl Hayden Bee Research Center (CHBRC) staff. 17g of each pollen sample was subjected to a full panel (204 compounds) test of pesticide residues by the National Science Laboratories, USDA-AMS, Gastonia, NC, USA, using the QuEChERS method [58]. A list of all of the pesticides tested and their limit of detection is given in S1 File.

The source of the pollens was investigated using ITS DNA sequencing and microscopy. The ITS DNA sequence was determined for each of the samples based on the methods of Little et al. [59] and Wilson et al. [60] using a sample of 30mg of each ground pollen. The eluted DNA was subjected to PCR using the ITS primers and PCR protocol described by Little et al. [59] in a 25μl reaction using GoTaq (Promega) according the manufacturer’s directions. The PCR product was visualized on a 1% agarose gel stained with SYBR Safe DNA gel stain (ThermoFisher), cut out, purified and cloned. 100–110 colonies were picked and subjected to a PCR with M13F (5’-CGCCAGGGTTTTCCCAGTCACGAC-3’) and M13R (5’-TCACACAGGAAACAGCTATGAC -3’) primers. The PCR product was directly sequenced at UAGC (http://uagc.arl.arizona.edu/). The vector was removed from the resulting sequence and the sequence was characterized using the NCBI BLAST algorithm (Altschul et al. 1990). Representative ITS sequences were deposited in the NCBI nucleotide database under accession numbers MG650259-MG650287. Light microscopy was conducted at the Texas A&M University Palynology Laboratory. The pollens were first incubated with glacial acetic acid for 3.5 hours and then acetolyzed (1 part sulfuric acid: 9 parts acetic anhydride) with heat (80°C) for 10 minutes. The pollen was then rinsed in glacial acetic acid then 3 times with dH_2_O, and ethanol was added. Three sub-samples were taken from the tube, combined, and the ethanol was removed. 10–12 drops of glycerin were added and the sample was mixed thoroughly. A small drop of this mixed sample was mounted onto a 75 x 25 mm slide with a glass coverslip and examined at 400x and 1000x using an Olympus BX41 microscope. A minimum of 210 pollen grains were classified for each of the samples.

### Pollen nutrient analyses

The soluble protein content of 1g of each type of ground pollen was measured using a BCA Assay Kit (Thermo Fisher Scientific) according to the manufacturer’s directions. Amino acid content was examined at the Proteomics and Mass Spectrometry Facility at the Danforth Plant Science Center. The samples were transferred to glass vial inserts and incubated with 200μl of performic acid for 30 min at room temperature in the dark. The samples were then dried with a Speed Vac and hydrolyzed with 0.5% phenol/6M HCl under vacuum. The samples were dried again and resuspended in 100μL 20mM HCl and the amino acid content was analyzed using Ultra-Performance Liquid Chromatography with the AccQ-Tag Ultra Derivatization Kit (Waters). The column and solvents were also purchased from Waters. The samples were initially diluted by 10 and further dilutions or concentrations were made to bring the signal within the linear range of the assay; these adjustments were accounted for in the final calculations. 1μL of the reaction was injected. The concentration of each amino acid was determined (pmol/μL) against a series of standards run before the samples.

The content of fatty acids and sterols in each pollen type was analyzed at the Proteomics and Mass Spectrometry Facility at the Danforth Plant Science Center. The content of individual fatty acids was assayed using FAME analysis. The samples were weighed and dispensed into a screw top glass tubes. 2.5 mL of 2.5% H_2_SO_4_ in methanol, 25 μL of BHT (0.2%) and 30 μL of C17:0 TAG (10mg/ml) was added. The samples were heated at 85°C for 2 hours. Fatty acid methyl esters were extracted with hexane and analyzed with a Thermo Trace GC-FID using a 0.25μm x 30 M HP Innowax column. The content of the sample oil was calculated based on the internal standard 17:0 TAG. Final oil content was normalized by the initial weight of the sample. For sterol analysis, 100mg of each pollen was extracted with 400μL hexane. The samples were homogenized in a Tissue Lyser II at 20 Hz for 10 min. Solids were collected by centrifugation and 1μl of each was analyzed with GC-MS using a Thermo Trace Ultra/ITQ 900 and a 0.25μm x 30 M Phenomenex ZB5-MSi column. The concentrations of the sterols that were detected were determined using a cholesterol standard curve for reference. Lastly, the total lipid (free fatty acid) content of each pollen was determined using a vanillin assay as described in [3, 61].

### Source bees

Five, 10 frame colonies headed by *Apis mellifera ligustica* queens from a commercial queen breeder in California supplied the bees for this experiment. The colonies were maintained for over a year at the CHBRC in Tucson, Arizona, USA and were compositionally equivalent. Sealed brood frames from these five colonies were placed into a hive box in a temperature-controlled room (33°C ± 1°C). All adults that emerged over an 18h period were collected for further use.

### Effects of different pollens on hypopharyngeal gland size

Emerged young adults (≤18h old) were placed into plexiglass cages (11.5cm × 7.5cm × 16.5cm) with wire screen on either side for air flow. Bees fed pollen were given distilled water, 50% sucrose (w/v), and one of the three types of loose pollen. The sugar, water, and pollens were provided *ad libitum*. Bees deprived of pollen were fed only 50% sucrose and water *ad libitum*. Five cages were constructed for each treatment, yielding a total of 20 cages (5 cages x 4 treatments). Each cage contained 100 bees. Dead bees were removed each day; fewer than 5 bees died within each cage over the course of the experiment. Consumption was measured for each of the cages supplied with pollen as the total grams of pollen consumed by the bees in the cage over 8d.

The HGs of bees fed each diet were measured to investigate whether pollen type impacted HG size and to determine the nutritional value of each pollen. At 8d of age, 10 bees were collected from each cage and flash frozen in liquid nitrogen and maintained at -70°C until their glands were measured. For each bee, the HGs were dissected into a PBS buffer and between 10 and 24 (average = 14) randomly selected acini per gland were visualized at 6X magnification. The area of the acini (mm^2^) was measured using the Leica Applications Suite v.3.8.0 software. The nutritional value of each pollen was assessed in three ways: (1) by comparing the HG sizes of bees fed either of the four diets (3 pollens or no pollen), (2) by comparing HG size differences for bees fed either of the three pollen types, controlling for differences in consumption, and (3) for each of the three pollen types, regressing the mean acinus size for each cage against the average amount of pollen consumed by the bees in that cage. In the first comparison, we analyzed the data for HG size of bees fed the four diets. For this dataset, the average acinus size for each bee was calculated and square root transformed to improve normality. The transformed data were analyzed using an ANOVA where pollen type, cage, and pollen type x cage were the independent variables. Pairwise differences between each pollen type and the no pollen treatment were evaluated using a Tukey’s HSD test. Because consumption might impact gland size, we next focused on just the bees fed pollen and performed the second comparison listed above. We tested whether pollen type, consumption, and consumption x pollen type significantly impacted the average cage-wide acinus measurement. The data were normally distributed and were analyzed using an ANOVA. In the third comparison, as an additional *post hoc* way to determine the nutritional value of the different pollens, the mean cage-wide acinus size was regressed against the average amount of pollen consumed by the bees in each cage provided with pollen. The slope was calculated for each pollen type to provide an index for amount of HG growth each unit of consumed pollen provides [17].

### Natural pollen choice assays

Preference for one pollen over another was assayed through pairwise choice assays. Approximately 50 bees ≤18h old were set up in cages as described above. Sucrose (50% w/v), water, and pollen were provided *ad libitum*. The bees in each cage were provided with two pollen types: almond and desert, almond and SE, or desert and SE. The pollens were freshly ground upon removal from the -20°C freezer using a coffee grinder and were weighed prior to feeding. The bees were monitored for 8 days and fresh pollen was provided daily. Mortality was negligible in each cage (≤3 bees over 8d); dead bees were removed daily. Five cages were set up for the SE/desert and almond/desert assays. Ten cages were initially set up in two separate trials (performed at two different times) for the almond/SE cages, but only nine were monitored over the 8d period (one cage in the second trial failed due to a faulty sugar bottle). Pollen consumption was recorded daily for 8d and the total amount of each pollen type that was consumed by the bees in each cage was compared. A modified Levene’s test [26] was used to determine whether the one pollen type was preferred over the other.

### Supplemented pollen choice assays

In bumblebees, the ratio of protein to lipids is implicated in pollen choice [62], suggesting that they can detect the ratio of protein to lipids in pollen and consume more of these optimally balanced pollens accordingly. In the natural pollen choice assays, the desert pollens were consumed more than the SE pollen, and we wondered if this was because the desert pollen had a lower protein to lipid ratio (P:L) of 3.5 than SE pollen (P:L = 6.27). To address this, we performed a series of choice experiments using supplemented and natural pollens. We hypothesized that if nurse bees detect the P:L ratios of pollens and choose pollens based on their nutritional value, they will preferentially consume pollens with the P:L = 3.5. We tested this hypothesis in three ways. First, we asked whether SE pollen could be made as equally attractive as the natural desert pollen by adding lipids and reducing its P:L ratio to 3.5 (Experiment 1 in Table 1). Next, we asked whether supplemented SE pollen could be made more attractive than the natural SE pollen by adding lipids and decreasing its P:L ratio to 3.5 (Experiment 2 in Table 1). Last, we asked whether we could increase the nurses’ preference for the SE pollen by decreasing its P:L ratio while keeping the concentration of lipids and proteins in the two diets equal (Experiment 3 in Table 1). This last comparison was made to separate the effect of the concentration of lipids and proteins from the actual P:L ratio. If a certain P:L ratio is detected and preferred by nurse bees, experiment 1, 2, and 3 should hold true to the expected outcomes indicated in Table 1. If only lipid content is detected and preferred by nurse bees, only experiment 2 should hold true. If non-nutritional factors impact pollen consumption, none of the experiments will show the expected results.

**Table 1:** Protein and lipid content of supplemented and natural, un-supplemented pollens (shaded) used in the supplemented pollen choice assays.

Experiment (expectation)	Pollen A	Protein (mg/g pollen)	Lipid (mg/g pollen)	P:L	Pollen B	Protein (mg/g pollen)	Lipid (mg/g pollen)	P:L
1 (pollen A = pollen B)	Supplemented SE	414	118.3	3.5	Natural desert	326	93	3.5
2 (pollen A > pollen B)	Supplemented SE	414	118.3	3.5	Natural SE	414	66	6.27
3 (pollen A = pollen B)	Supplemented SE	414	118.3	3.5	Supplemented desert	414	118.3	3.5

Natural pollens were supplemented with either protein (bovine casein (Sigma)) or lipids (soy lecithin (Alfa Aesar)) as in [62, 63]. The final concentration of the proteins and lipids and the protein to lipid ratios (P:L) of these pollens in each choice experiment is indicated in Table 1. Supplemented SE pollen was made by mixing 52.3 mg/g of lecithin and 30% w/v of sucrose with the SE pollen. This yielded a mix containing 414 μg of protein and 118.3 μg of lipids per mg of pollen and a P:L ratio of 3.5. Supplemented desert pollen was made by adding 88 mg/g of casein, 25.3 mg/g of lecithin, and 50% w/v of 30% sucrose into the desert pollen. This yielded a mix containing 414 μg of protein and 118.3 μg of lipids per mg of pollen and a P:L ratio of 3.5.

Thirty cages containing ~50 newly emerged workers were constructed. The bees in these cages were presented with the following choices: supplemented SE versus natural desert pollen (N = 10 cages; Hypothesis 1), supplemented SE versus natural SE pollen (N = 10 cages; Hypothesis 2), or supplemented SE versus supplemented desert pollens (N = 10 cages; Hypothesis 3). Consumption of the pollens was measured after seven days. The mean consumption of each diet was compared using a modified Levene’s test.

### Liquid diet choice assays

Honey bees and bumblebees consume liquid diets that optimize their survival [57, 63]. Because HG development requires dietary protein and lipids, we tested whether nurse aged bees preferred certain ratios of protein and lipid (P:L ratio). Liquid diets were comprised using bovine casein (Sigma) as the protein source or soy lecithin (Alfa Aesar) as the lipid source as in [63]. P:L ratios were either 2.6:1, 3.5:1, or 6.27:1 to reflect the ratios observed in the almond, desert, and SE pollens, respectively. Thirty cages containing 50–100 newly emerged workers were constructed as described above. Bees were provided with water and 50% w/v sucrose *ad libitum*. The bees were also provided with two liquid diets that contained 30% w/v sucrose and dissolved casein and lecithin in the following ratios: 3.5:1 P:L and 2.6:1 P:L (N = 10 cages), 2.6:1 P:L and 6.27:1 P:L (N = 10 cages), and 3.5:1 P:L and 6.27:1 P:L (N = 10 cages). Liquid diets were provided *ad libitum*. Total consumption of either liquid diet was measured after one week. Differences in total consumption were compared using a modified Levene’s test. Cages with significant mortality (<30 bees alive after one week) were excluded from further analyses.

## Results

### Pesticide analysis

The almond, desert, and SE pollens were free of or exhibited trace amounts of virtually all of the pesticides tested for (Table 2). SE pollen had low amounts of DMPF, a product of Amitraz (a miticide) breakdown, and fluvalinate that were close to the limits of detection (LOD). The desert pollen contained the herbicide pendimethalin that was also close to the LOD. The almond pollen had DMPF, chlorpyrifos (an insecticide), and oxyfluorfen (an herbicide) that were close to the LOD. Almond pollen also had levels of cyprodinil (a fungicide), esfenvalerate (an insecticide), and pendimethalin (an herbicide) that were present in three to four times the LOD.

**Table 2:** Pesticide content (ppb) of the three pollens.

Pesticide	LOD (ppb)^A^	Southeast	Desert	Almond
2,4 Dimethylphenyl formamide (DMPF)	5	9	- ^B^	7
Carbendazim (MBC)	5	Trace^C^	Trace	-
Chlorpyrifos	5	Trace	-	7
Coumaphos	3	-	-	Trace
Cyprodinil	10	-	-	40
Esfenvalerate	5	-	-	14
Fluopyram	5	-	-	Trace
Fluvalinate	5	9	-	-
Imidacloprid	6	-	Trace	-
Metalaxyl Total	5	Trace	-	-
Methoxyfenozide	5	-	-	Trace
Oxyfluorfen	5	-	-	7
Pendimethalin	15	-	15	56
Piperonyl butoxide	15	-	Trace	-
Propargite	15	Trace	-	Trace
Pyraclostrobin	5	-	Trace	-
Thymol	50	Trace	Trace	Trace
Trifluralin	5	Trace	-	-

^A^ Limit of detection in parts per billion (ppb).

^B^ “-”= Not detected.

^C^ Trace values are >0 but too close to the limits of detection to be reliably determined.

### Pollen plant type analysis

Light microscopy revealed that the almond, desert, and SE pollens contained grains from 1, 9, and 11 plant taxa, respectively (Table 3). Almond was therefore the least taxon rich sample of pollen, while desert and SE were comparable. The pollens were also comprised of grains from different plant taxa. All of the almond pollen grains were from plants of the genus *Prunus*; almond is *Prunus dulcis*. SE pollen contained mostly grains from the Brassicaceae (38%), *Vicia* sp. (vetch; 21%), *Trifolium*/*Melilotus* sp. (clover; 12%), and *Artemisia* sp. (wormwood or sage; 7%). Desert pollen contained grains from *Fraxinus* sp. (ash; 45%), *Prosopis* sp. (mesquite; 19%), *Pistacia* sp. (Chinese pistache; 11%), Brassicaceae (10%), and *Eucalyptus*/*Melaleuca*/*Eugenia* sp. (8%). All other taxa were ≤ 5% abundance.

**Table 3:** Diversity of pollen grains in pollens based on light microscopy and ITS sequencing.

Plant taxon	Almond		Southeast		Desert	
	% ITS	% microscopy	% ITS	% microscopy	% ITS	% microscopy
Amaranthaceae (amaranth & goosefoot)	0	0	0	5	0	0
*Artemisia* (sagebrush)	0	0	0	7	0	0
Asteraceae (dandelion-type)	0	0	0	4	0	0
Asteraceae (sunflower-type)	0	0	8	1	0	0
*Astragalus* (milk vetch)	0	0	0	0.4	0	0
Brassicaceae (mustard family)	0	0	3	38	1	10
Caryophyllaceae	0	0	0	1	0	0
*Centaurea* (thistle)	0	0	0	1	0	0
*Cirsium* (thistle)	0	0	0	2	0	0
*Dalea* (prairie clover)	0	0	0	0.4	0	0
*Eucalyptus* (gum)	0	0	0	0	0	8
Fabaceae (legumes)	0	0	0	0	0	2
*Fraxinus* (ash)	0	0	0	0.4	31	45
*Leucaena* (leadtree)	0	0	0	0	0	0.5
*Leucophyllum* (cenizo, sage)	0	0	0	0	0	1
*Larrea* sp. (creosote)	0	0	0	0	31	0
*Phoradendron* sp. (mistletoe)	0	0	0	0	35	0
*Pistacia* (pistacia)	0	0	0	0	0	11
*Platanus* (sycamore)	0	0	0	0	0	3
*Prosopis* (mesquite)	0	0	0	0	1	19
*Prunus* sp.	100	100	4	0	0	0
Rosaceae (rose family)	0	0	0	6	0	0
*Salix* (willow)	0	0	0	0.4	0	0
*Trifolium/Melilotus* (clover)	0	0	0	12	0	0
*Vicia* (vetch)	0	0	77	21	0	0
*Caltha* sp.	0	0	7	0	0	0
*Citrus* sp.	0	0	1	0	0	0
Unknown pollen	0	0	0	0.4	0	1
Sequences or grains examined	85	210	73	255	83	219
Taxa observed	1	1	6	11	5	9

The ITS sequencing revealed slightly different patterns than the light microscopy (Table 3). The almond pollen contained 11 poor quality sequences and 85 good quality sequences that could be identified by sequence similarity to the NCBI nucleotide database. All sequences were from *Prunus* sp. The SE pollen had a large number of sequences that were either poor quality or unidentifiable (23 sequences) or that matched to fungal sequences in the NCBI nucleotide database (5 sequences). The 73 SE pollen sequences that remained had a large number of *Vicia* sp. (vetch; 77%) as well as Asteraceae (sunflower; 8%), Brassicaceae (mustard; 3%), *Prunus* sp. (4%), *Caltha* sp. (buttercup; 7%), and *Citrus* sp. (1%) sequences. Thirteen of the desert sequences were poor quality and could not be used for further analysis. The remaining 83 sequences were from a variety of desert plants, including *Larrea* sp. (creosote; 31%), *Phoradendron* sp. (desert mistletoe; 35%), and *Prosopis* sp. (mesquite; 1%). Similar to what was found with the microcopy, the ITS sequence-based data showed that almond was the least taxon rich sample of pollen (1 taxon), while desert and SE were comparable and were from 5 and 6 plant taxa, respectively.

**Fig 1:**
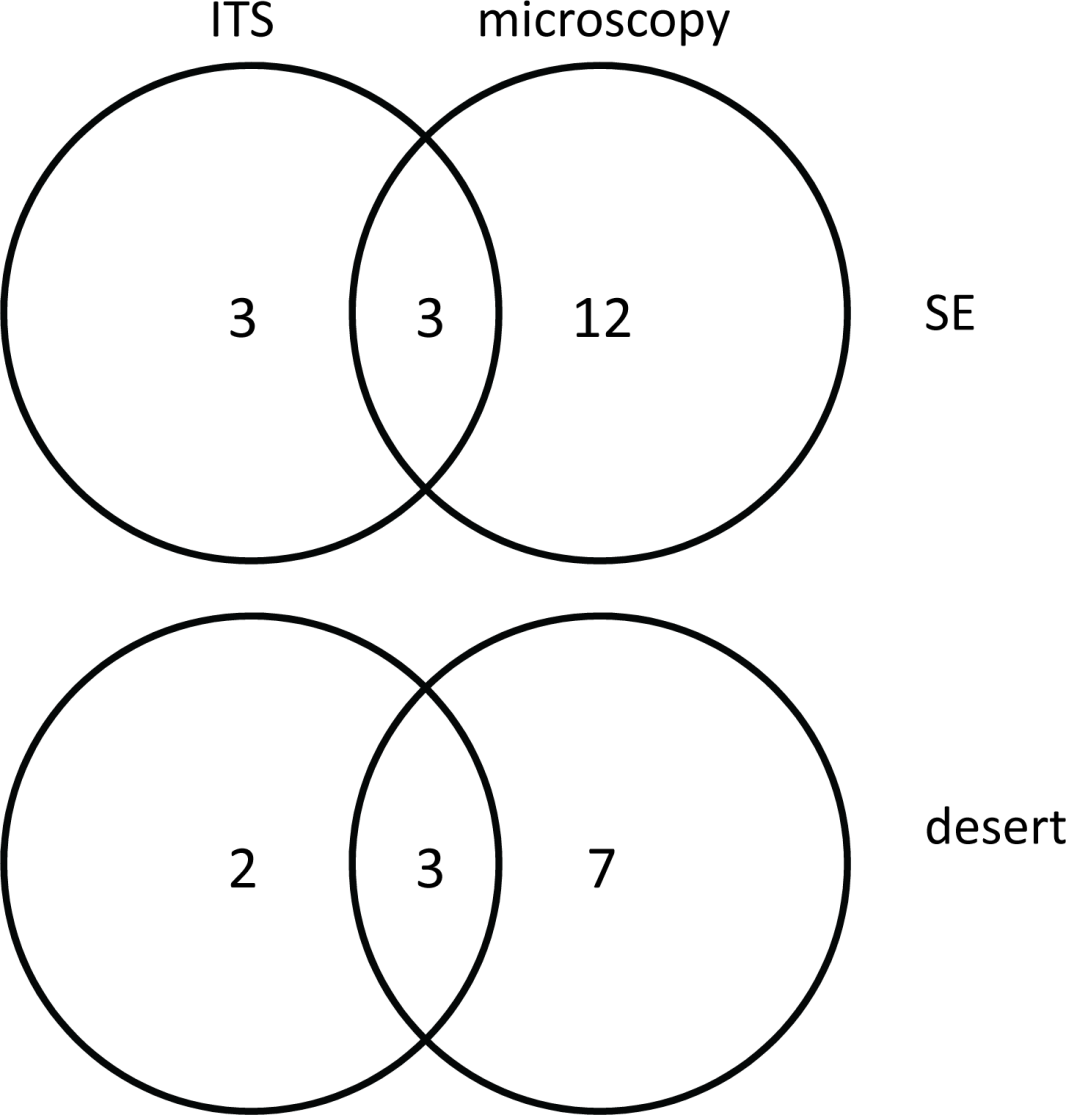
Venn diagram representing the types of plant taxa identified in honey bee-collected pollens. The number of plant taxa from pollens from the Southeastern United States (“SE”) and the desert are shown and were catalogued using ITS sequences and light microscopy.

In some cases, the taxa found in using one method were not found using the other method (Fig 1). For example, for the SE pollen, the ITS dataset contained sequences from *Caltha* sp., *Prunus* sp., and *Citrus* sp. but the light microscopy did not. Conversely, the light microscopy found twelve taxa not seen in the ITS dataset. The two methods were both successful at finding grains from Brassicaceae, *Vicia* sp., and Asteraceae (sunflower-type). For the desert pollen, the two methods detected *Fraxinus* sp., *Prosopis* sp., and Brassicaceae pollens. Only ITS detected *Phoradendron* sp. and *Larrea* sp. pollens, and only microscopy detected *Eucalyptus* sp., *Platanus* sp., Fabaceae, *Leucophyllum* sp., and *Leucaena* sp. pollens.

### Pollen nutrient analyses

The SE pollen (414 mg/g of pollen) had the highest amount of total soluble protein, followed by the desert (326 mg/g of pollen) and almond (283 mg/g of pollen) pollens. The lipid content was highest in the almond pollen (109 mg/g of pollen), followed by the desert (93 mg/g of pollen) and SE (66 mg/g of pollen) pollens. This yielded a protein:lipid (P:L) ratio that was highest in the SE pollen (P:L = 6.27), followed by the desert (P:L = 3.50) and almond (P:L = 2.60) pollens.

The amino acid profile of the three pollens differed (Table 4). Almond pollen had the greatest amount of lysine, threonine, valine, glycine, and proline. Desert pollen was highest in histidine, methionine, phenylalanine, arginine, cysteine, and tyrosine. SE pollen was highest in isoleucine, leucine, serine, alanine, aspartate, and glutamate.

**Table 4:** Amino acids present in each pollen type.

Amino acid (ug/ml)	Almond	Desert	Southeast
*Essential*			
Histidine	9.448486	13.67088	6.622645
Isoleucine	14.48419	13.24066	14.86945
Leucine	21.34955	19.87424	21.47902
Lysine	12.10468	5.759523	11.7987
Methionine	15.14506	18.47667	9.88756
Phenylalanine	21.0837	24.94782	18.57727
Threonine	13.63694	13.39537	13.5351
Valine	17.97619	16.11309	17.06994
*Conditional*			
Arginine	16.13717	18.10809	14.2396
Cysteine	6.822342	10.07631	8.243658
Glycine	16.668	14.86437	16.23712
Proline	30.14533	23.70501	16.17188
Serine	12.14139	12.35337	12.37291
Tyrosine	5.942106	6.386745	4.657655
*Nonessential*			
Alanine	12.34408	10.10775	12.70072
Aspartate	14.06422	13.22061	14.20677
Glutamate	17.53759	17.20463	17.69752

The fatty acid profile of the pollens also differed (Table 5). SE pollen was highest in myristic (14:0), palmitic (16:0), stearic (18:0), α-linolenic (18:3), arachidic (20:0), erucic (22:1), and cervonic (22:6 (n-3)) acids but lowest in palmitoleic (16:1), margaric (17:0), oleic (18:1 (n-9)), linoleic (18:2), behenic (22:0), and lignoceric (24:0) acids. Almond pollen was highest in behenic acid (22:0) and lignoceric acid (24:0) and lowest in palmitic (16:0), stearic (18:0), vaccenic (18:1 (n-7)), and erucic (22:1) acids. Desert pollen was highest in palmitoleic (16:1), margaric (17:0), oleic (18:1 (n-9)), vaccenic (18:1 (n-7)), linoleic (18:2), and behenic (22:0) acids and lowest in myristic (14:0), α-linolenic (18:3), and arachidic (20:0) acids. Total omega fatty acid content was highest in the almond pollen and lowest in the SE pollen. The omega-6:omega-3 ratio was highest in the desert pollen and lowest in the SE pollen.

**Table 5:** Fatty acids (FAs) present in the different pollen types.

Common name	FA name	Southeast	Almond	Desert
Myristic acid	14–0	2.0^A^	0.1	0
Palmitic acid	16–0	20.0	16.8	18.3
Palmitoleic acid	16–1	0	0.4	0.5
Margaric acid	17–0	13.8	17.5	18.0
Stearic acid	18–0	2.3	1.1	1.4
Oleic acid	18–1 (n-9)	4.0	4.2	6.7
Vaccenic acid	18–1 (n-7)	0.5	0.4	0.5
Linoleic acid^B^	18–2	9.8	30.9	33.5
α-Linolenic acid^C^	18–3	32.3	23.0	16.7
Arachidic acid	20–0	0.8	0.8	0.2
Gondoic acid	20–1	0	0	2.1
Behenic acid	22–0	0	1.7	0.3
Erucic acid	22–1	3.7	0	0.5
Cervonic acid	22–6 (n-3)	1.8	0	0
Lignoceric acid	24–0	0	1.3	0.6

^A^ Each value is represented as the percent of total area under the curve generated by GC-MS FAME analysis and represent relative amounts.

^B^ Omega-6 fatty acid.

^C^ Omega-3 fatty acid.

Seven sterols could be reliably identified based on their retention times (Table 6). Almond pollen was highest in stigmasterol, γ-sitosterol, and rubrosterone and lowest in 29-methylisofucosterol. SE pollen was highest in 24-methylenecholesterol, cholest-23-ene, 9,19-cyclochloestene-3,7-diol, 4,14-dimethyl-, 3-acetate, and 29-methylisofucosterol. Desert pollen had a low relative abundance of almost all of the sterols detected except for rubrosterone.

**Table 6:** Sterol content of the different pollens.

			Pollen		
Name	Chemical formula	Retention time	Almond	Southeast	Desert
Stigmasterol	C_29_H_48_O	9.12	0.00025 ^A^	0.00010	1.3 x 10^−5^
24-methylenecholesterol	C_28_H_46_O	9.21	1.4 x 10^−5^	1.4 x 10^−5^	N.D.
5β -Cholest-23-ene	C_27_H_46_	9.36	N.D.	1.7 x 10^−5^	N.D.
9,19-cyclochloestene-3,7-diol, 4,14-dimethyl-, 3-acetate	C_31_H_52_O_3_	9.56	N.D.	4.1 x 10^−5^	N.D.
γ-sitosterol	C_29_H_50_O	9.66	1.0 x 10^−5^	N.D.	N.D.
29-methylisofucosterol	C_30_H_50_O	9.77	1.4 x 10^−5^	4.3 x 10^−5^	1.5 x 10^−5^
Rubrosterone	C_19_H_26_O_5_	11.30	4.7 x 10^−5^	1.2 x 10^−5^	1.4 x 10^−5^

^A^ Each value is represented as the percent of total area under the curve and represent relative amounts.

### Hypopharyngeal gland (HG) sizes by pollen type

**Fig 2:**
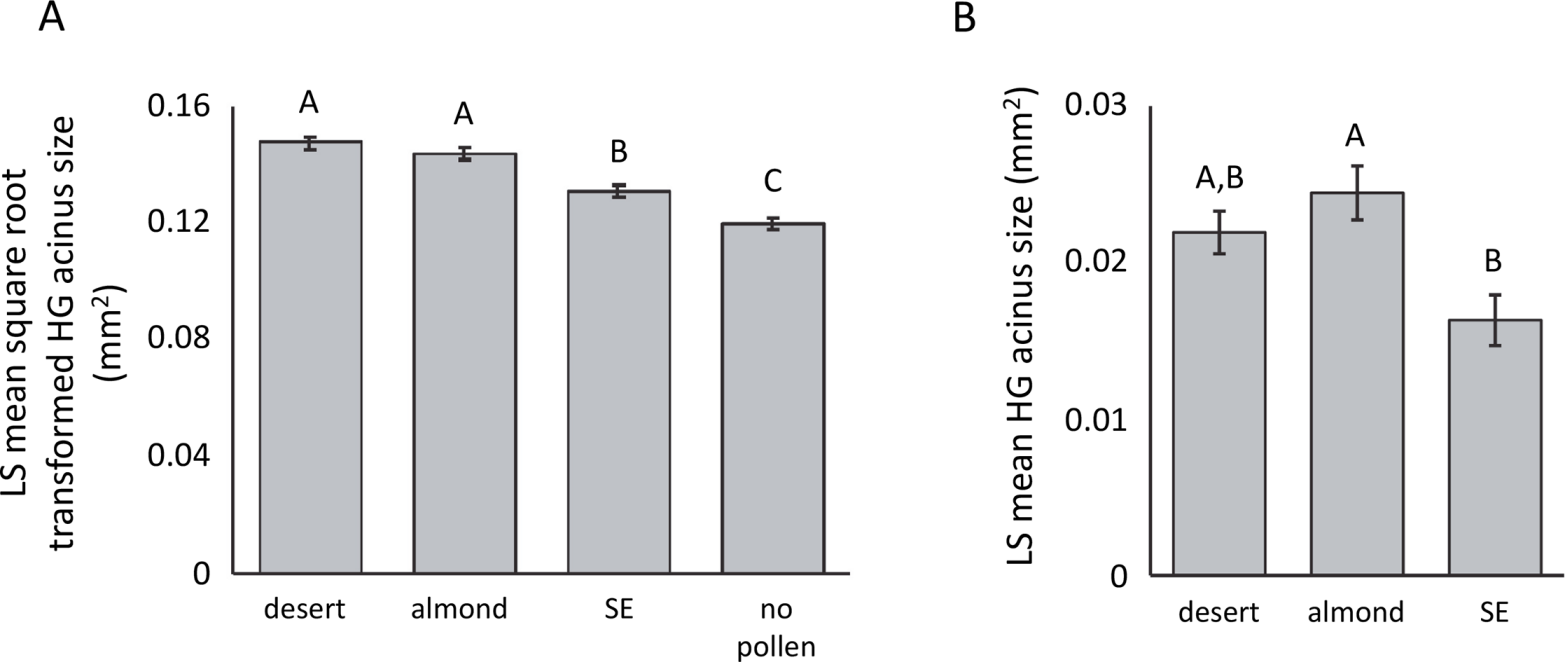
**Hypopharyngeal gland (HG) sizes differed according to diet when consumption was not (A) or was (B) accounted for.** Gland size is represented by the least square (LS) mean estimate of (A) the square root transformed average acinus size (mm^2^) or (B) the average acinus size (mm^2^) relative to pollen type (see methods). Bees were fed for 8d on each of the three diets. “SE” indicates pollen from the Southeastern United States. Bars with different letters are significantly different (p<0.05). Error bars represent S.E. around the LS mean values.

Bees fed either the desert (average HG acinus size = 0.03 mm^2^ ± 0.001 S.E.) or almond pollen (average HG acinus size = 0.021 mm^2^ ± 0.001 S.E.) had the largest HGs, followed by bees fed SE pollen (average HG acinus size = 0.017 mm^2^ ± 0.0005 S.E.). Bees fed no pollen had the smallest HG acinus size (average HG acinus size = 0.014 mm^2^ ± 0.0005 S.E.). Hypopharyngeal gland size differed according to pollen type (Fig 2). Pollen type (F_3,180_ = 35.45, p<0.0001, Fig 2A), cage (F_4,180_ = 16.11, p<0.0001), and the pollen type x cage (F_12,180_ = 11.11, p<0.0001) interaction significantly impacted HG size for the data set that included bees fed no pollen (i.e., the first comparison outlined in the methods). The desert and almond pollens resulted in a similar increase in HG size over those from bees fed no pollen. The SE pollen provided a smaller increase in HG size over those from bees fed no pollen. For the data set summarizing the HG sizes for only bees fed pollen (i.e., the second comparison outlined in the methods), pollen type (F_2,9_ = 6.40, p = 0.018; Fig 2B) and consumption (F_1,9_ = 21.17, p = 0.001) significantly impacted the cage-wide HG acinus sizes. The interaction between consumption and diet did not impact HG size. Accounting for consumption, the almond pollen conferred the greatest amount of HG growth and was significantly higher than the SE pollen, which provided the least nutrition. The desert pollen provided a medium level of nutrition that was not significantly different from the almond or SE pollens. The slope of the average acinus size per cage regressed against the average amount of pollen consumed by the bees in that cage provided a nutritional index for each pollen (i.e., the third comparison outlined in the methods). The almond and desert pollens yielded roughly equal HG growth per unit of pollen consumed (almond pollen slope = 0.0063; desert pollen slope = 0.0062). The SE pollen yielded the least amount of growth per unit of consumption (SE pollen slope = 0.0031).

### Natural pollen choice assays

**Fig 3:**
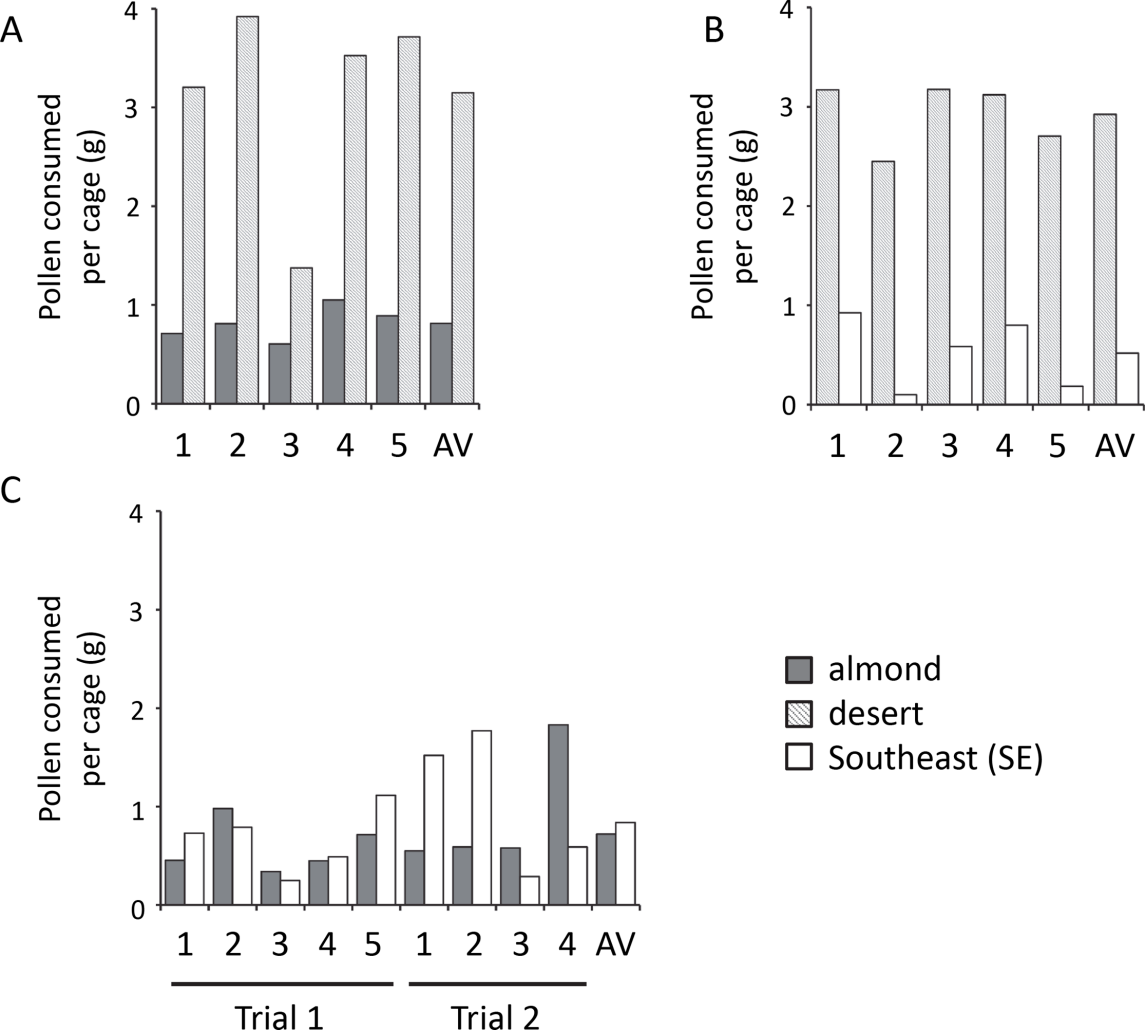
Pollen (g) consumed by bees in each cage in choice assays. Consumption is shown for each cage for pairwise comparisons between (A) almond and desert, (B) desert and SE, or (C) almond and SE pollens. Average (AV) consumption over all cages is also shown. For each panel, both types of pollen were provided *ad libitum* and the total amount of pollen consumed over 7d was recorded. For panels A and B, there were significant differences in consumption (p<0.001). In panel C, consumption did not differ. Experiments in panel C were conducted in two different trials at two different times.

Three types of choice cages were constructed: almond versus desert pollen, almond versus SE pollen, and desert versus SE pollen. Overall, the bees consumed more pollen from the cages where almond and desert pollens were provided (average total amount consumed = 3.96 g ± 0.51 S.E. per cage) and where SE and desert pollens were provided (average total amount consumed = 3.44 g ± 0.30 S.E. per cage) compared to the cages where the SE and almond pollens were provided (average total amount consumed = 1.56 g ± 0.23 S.E. per cage). In the cages where bees were presented with almond or desert pollen (N = 5 cages), the bees consumed more desert pollen on average (|t_4_| = 11.62, p = 0.0003; Fig 3A). When given a choice between desert and SE pollen, the bees consumed more desert pollen compared to SE pollen (|t_4_| = 9.75, p = 0.0006; Fig 3B). When presented with almond and SE pollen (N = 9 cages), the bees preferred the SE pollen in 5 cages and the almond pollen in 4 of the cages (Fig 3C) and the average amount of either type of pollen consumed per cage was not statistically different.

### Supplemented pollen choice assays

**Fig 4:**
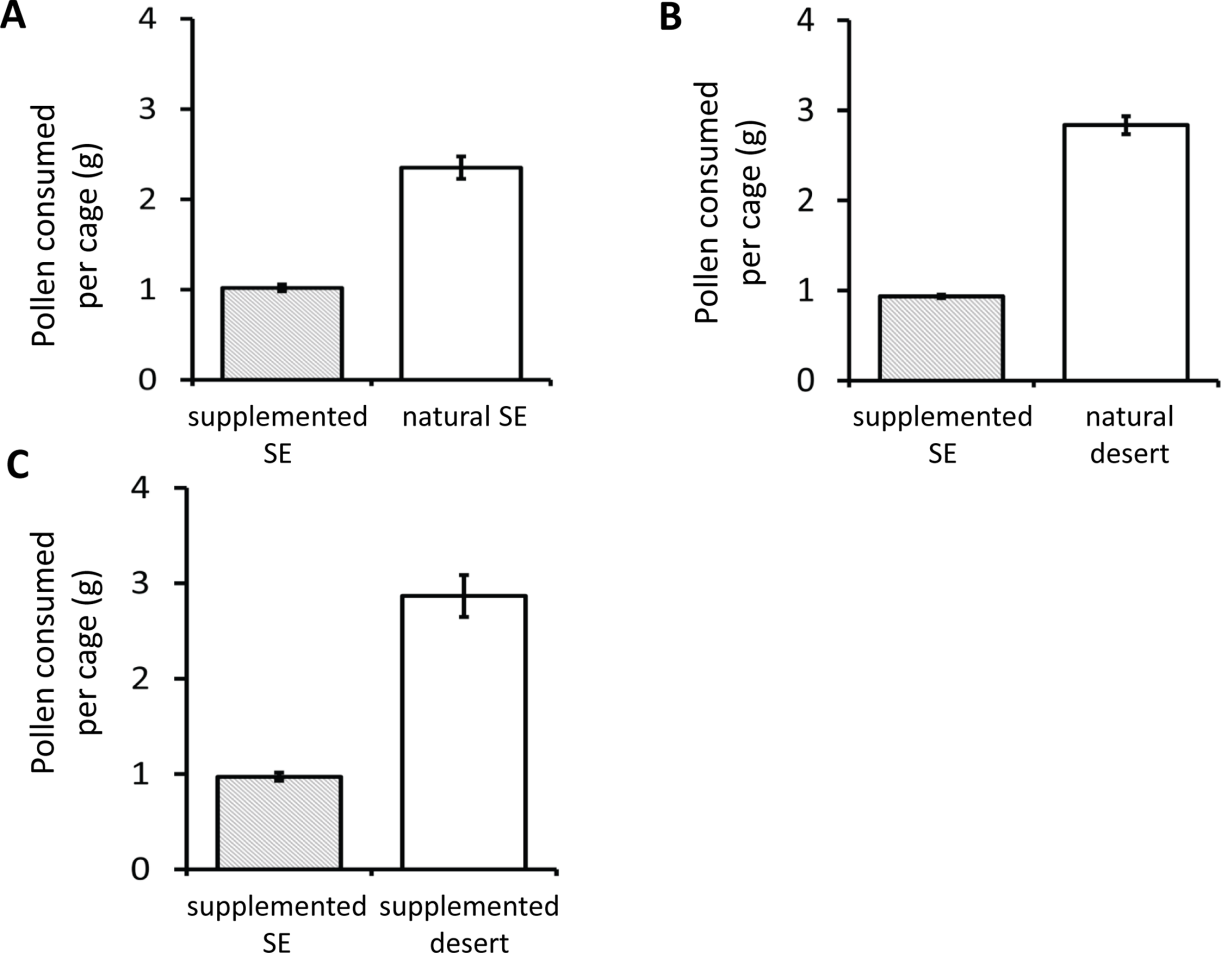
Average pollen (g) consumed by bees in each in supplemented pollen choice assays. Both types of pollen were provided *ad libitum* and the total amount of pollen consumed over 7 days was recorded. The error bars represent the S.E. around the mean consumption. Significant differences (p<0.0001) were observed in all comparisons. “SE” indicates pollen from the Southeastern United States.

In the cages presented with supplemented SE pollen and natural SE pollen, the bees preferred the natural SE pollen (average consumption of natural SE pollen = 2.35 g ± 0.12 S.E., average consumption of supplemented SE pollen = 1.02 g ± 0.04 S.E.; |t_9_| = 13.35, p<0.0001; Fig 4A). This suggested that the added casein and lecithin may have been distasteful for the bees. In the cages presented with natural desert pollen and supplemented SE pollen, the bees preferred the natural desert pollen (average consumption of natural desert pollen = 2.84 g ± 0.10 S.E., average consumption of supplemented SE pollen = 0.94 g ± 0.02 S.E.; |t_9_| = 33.88, p<0.0001; Fig 4B). In the cages provided with the supplemented SE and supplemented desert pollens, the bees preferred the supplemented desert pollen (average consumption of supplemented desert pollen = 2.87 g ± 0.22 S.E., average consumption of supplemented SE pollen = 0.97 g ± 0.04 S.E.; |t_9_| = 13.00, p<0.0001; Fig 4C). Therefore, pollen choice was not greatly influenced by manipulating the P:L ratio.

### Liquid diet choice assays

For the 8 surviving cages fed the 2.6:1 P:L and 6.27:1 P:L diets, each cage consumed an average of 2.38 g of diet, 1.29 g ± 0.21 S.E. of the 2.6:1 diet and 1.09 g ± 0.06 S.E. of the 6.27:1 diet. For the 8 surviving cages fed the 3.5:1 P:L and 2.6:1 liquid diets, each cage consumed an average of 2.08 g of diet, 0.96 g ± 0.07 S.E. of the 3.5:1 diet and 1.12 g ± 0.10 S.E. of the 2.6:1 diet. For the 7 surviving cages fed the 3.5:1 P:L and 6.27:1 P:L diets, each cage consumed an average of 2.20 g of diet, 1.02 g ± 0.04 of the 3.5:1 diet and 1.18 g ± 0.20 S.E. of the 6.27:1 diet. Consumption of either liquid diet was not significantly different for any of the comparisons.

## Discussion

We catalogued the nutritive properties and plant taxon richness of three different honey bee collected pollens (almond, desert, and Southeast/SE pollen). We asked whether certain pollens promoted greater HG growth than others and whether nurse bees preferred pollens that promote gland growth. We also conducted choice assays with supplemented pollens and liquid diets to test whether choice was influenced by the content and ratios of protein and lipids, as is it is in bumblebees [62, 63]. The three pollens varied in their lipid and protein content, their fatty acid, sterol, and amino acid profiles, and the number of plant taxa found in the pollens. Bees tended to have larger glands when fed almond or desert pollens compared to SE pollen, and these nutritious pollens contained higher amounts of certain amino acids and fatty acids and lower P:L ratios. HG gland size was not influenced by the number of plant taxa in the pollen sample. In paired choice assays with natural un-supplemented pollen, nurses preferred the nutritious or taxon rich pollens. In these natural pollen choice assays, we noted that the consumption of the desert pollen was rather high compared to the other two types of pollens. To more closely examine the question of whether desert pollen was simply more attractive or whether nutrition influenced pollen choice, we performed two additional choice experiments using either supplemented pollens or liquid diets that mimicked the P:L ratios of the natural pollens. Supplementing natural pollens with protein or lipids made undesirable pollens only slightly more desirable. Also, nurses prefer any of the liquid diets. In sum, we find that nutritional value varies among pollen types, nutritional value (as measured by HG size) may be related to the presence of certain nutrients in the pollens but not the taxon richness, and that nutritional value plays a minimal role in nurse bee diet choice.

Both ITS sequencing and light microscopy showed that the three pollens differed in the number and type of plant taxa. The two methods used to assay the types of plant taxa contributing to the pollen grains differed. Differences are not uncommon, but usually fall in the other direction, with greater diversity detected using ITS barcoding [28, 29, 64]. It may be that the ITS clone libraries did not adequately sample the taxa present in the sample. Taxon richness increased in the same fashion regardless of methodology, with the most taxa found in the SE pollen, slightly fewer taxa in the desert pollen, and substantially fewer taxa in the almond pollen. That the almond pollen was so homogenous is unsurprising given that it was collected from hives during almond pollination. Even when other plants are blooming, honey bee visitation to almond blooms is not reduced [65]. In addition to differences in the number of taxa present in the pollen mixtures, the type of plants comprising the pollens differed tremendously. None of the plant taxa from one pollen type was found in the other two. This was also expected, because the pollens were sourced from very different areas of the United States.

Hypopharyngeal gland size differed according to diet, with the almond and desert pollens causing the most growth and the SE pollen causing the least amount of growth. Additionally, the slope of HG size regressed against consumption was highest in the desert and almond pollens, suggesting that these diets provided more nutrition per unit of consumption than the SE pollen. The pollens that were most nutritious (as defined by HG growth) were high in the amino acids histidine, methionine, proline, and tyrosine and palmitoleic, margaric, and linoleic fatty acids. Omar *et al*. [66] also found that pollens that stimulated HG growth were high in phenylalanine and arginine. Nutritious pollens also had lower P:L ratios, suggesting that lipids are important for HG development. Consistent with other studies [4, 6], we found that HG size is not positively related with total pollen protein content, suggesting that protein content alone is not the most important metric influencing HG size. In contrast to previous work [67], we did not find a relationship between α-linolenic acid and HG size.

We used a series of choice assays to test the hypothesis that nurse bees assess the nutritional value of pollens. In these choice assays, bees were presented with natural pollens that differed in nutrition and the bees’ consumption of either pollen was recorded. The bees preferred more nutritious pollens, but we also observed that bees consumed much more of the desert pollen overall, and so it was possible that they were simply more attracted to that pollen due to its non-nutritive phagostimulatory properties (i.e., microbes, secondary compounds, odors, or enzymes [39, 68–71]). To disentangle the effects of overall palatability and nutrition on choice, we conducted two additional choice experiments using supplemented or natural pollens and liquid diets as in [62, 63]. Natural and supplemented desert pollen was always more attractive than the supplemented SE pollen (Fig 4B and 4C), so simply adding lipid and/or protein could not totally reconcile the consumption differences between the SE and desert pollens. This may have been because the SE pollen was unattractive to the bees (Fig 3B). However, the supplement itself was not distasteful because supplementation did not impact the consumption of the desert pollen (Fig 4B and 4C). Although supplementation of the SE pollen did not increase its consumption relative to the natural and supplemented desert pollens, there was a greater difference in consumption when bees were provided with natural desert pollen and natural SE pollen (Fig 3B; 5.6 fold difference between pollen types) compared to when they were provided with natural desert pollen and supplemented SE pollen (Fig 4B; 3.0 fold difference between pollen types). This was also true when the desert pollen was supplemented (Fig 4C). So P:L ratios themselves, irrespective of total protein and lipid content, may exert a very subtle influence on nurse choice. With respect to the liquid diet choice assay, when nurse bees were presented with liquid diets containing P:L ratios equal to those found in natural pollens, they did not prefer one diet over the other. Although the results of the natural pollen choice, the supplemented pollen choice, and liquid diet choice assays are mixed, the overarching theme is that nutritional value of the diet influences choice only slightly, if at all. From these three choice experiments, we conclude that, unlike in other bees [32, 34, 36, 62, 63], the nutritional value of pollen did not impact its consumption, suggesting that nurse bees either cannot or do not assess pollen quality. Behmer [27] suggested that insect herbivores may not regulate the intake of nutrients if only small amounts are required and if they are abundant in forage. Perhaps a preference would have emerged if these nurses were under greater nutritional duress or if brood was present.

Several studies show that pollen diversity benefits honey bee health when the bee is immune challenged [5, 6, 11], but under healthy conditions the benefit of diversity is unclear. We did not observe a relationship between HG size and plant taxon richness. Bees fed almond pollen, the least taxon rich of the three pollens tested, had HGs that were equal in size to bees fed a taxon-rich diet of desert pollen. Bees fed almond pollen also had glands that were larger than bees fed SE pollen, which was approximately as diverse as the desert pollen. In choice assays, the bees preferred the more diverse pollens, but because of the results of the other choice experiments (see above) and because HG size was not correlated with taxon richness, the increased attractiveness of the taxon rich pollen is likely not a reflection of its nutritional value. Instead, the more taxon-rich pollens may have presented a wider array of phagostimulants that increased their consumption over the monoculture pollen. More work should be done to address the relationship of floral diversity and nutrition, however, as the number of pollens we tested was limited.

Recent studies show that honey bee foragers choose pollens based its nutritional value [44, 45]. If foragers choose pollens to “rescue” nutritional deficiencies, how do they know what pollen to choose? Nurse bees consume the vast majority of pollens and have more pollen-digesting proteolytic activity in their guts compared to foragers [46, 47] and experience the greatest cost if quality pollen is not plentiful or highly nutritious. If nurses communicate hive pollen deficiencies to foragers [49], we hypothesized that they must first be able to assess pollen quality in order to then send this signal. The data from our choice assays do not support this hypothesis: while certain pollens provided more nutrition, nurse bees did not prefer higher-quality pollens. It is therefore probably not the case that nurse bees communicate nutritional deficiencies to foragers so that they are more likely to collect higher quality pollens. Further work is needed to reconcile our result with the rescue effect observed in other studies [44, 45]. One possibility for the disconnect is that this behavior depends on the nutrient in question. While certain nutrients are present on the pollen surface, others are embedded deep within the pollen grain. This might be why honey bees do not forage for resources high in protein [38, 40, 43], which is accessible only through lysis of the pollen grain exine, but can forage for pollens rich in fatty acids, which are present on the grain’s surface [39, 45, 72, 73]. Unfortunately, foraging for resources based on the chemical profile of the pollen surface could disconnect the needs of the nurses from those of the foragers, as the nutrients most important to nurses are more abundant on the inside of the grain.

## Supporting information

S1 File Hypopharyngeal gland acinus sizes by pollen type and cage. (XLS)

S2 File Consumption of pollens by bees in each cage according to pollen type. (XLSX)

S3 File Lipid analysis of SE (labeled “corn”), almond, and desert (labeled “rapini”) pollens. (PDF)

## References

[1] Goulson D, Nicholls E, Botias C, Rotheray EL. Bee declines driven by combined stress from parasites, pesticides, and lack of flowers. Science. 2015;347(6229):1255957 Epub 2015/02/28. 10.1126/science.1255957 . 25721506

[2] Woodard SH. Bumble bee ecophysiology: integrating the changing environment and the organism. Current Opinion in Insect Science. 2017;22:101–8. 10.1016/j.cois.2017.06.001 28805631

[3] Vaudo AD, Tooker JF, Grozinger CM, Patch HM. Bee nutrition and floral resource restoration. Current Opinion in Insect Science. 2015;10:133–41. 10.1016/j.cois.2015.05.008. 29588000

[4] Standifer LN. A comparison of the protein quality of pollens for growth-stimulation of the hypopharyngeal glands and longevity of honey bees, *Apis mellifera* L. (Hymenoptera: Apidae). Insectes Sociaux. 1967;14(4):415–25. 10.1007/bf02223687

[5] Di Pasquale G, Alaux C, Le Conte Y, Odoux JF, Pioz M, Vaissiere BE, et al Variations in the availability of pollen resources affect honey bee health. PloS One. 2016;11(9):e0162818 Epub 2016/09/16. 10.1371/journal.pone.0162818 27631605 PMC5025243

[6] Di Pasquale G, Salignon M, Le Conte Y, Belzunces LP, Decourtye A, Kretzschmar A, et al Influence of pollen nutrition on honey bee health: do pollen quality and diversity matter? PloS One. 2013;8(8):e72016 Epub 2013/08/14. 10.1371/journal.pone.0072016 23940803 PMC3733843

[7] Haydak MH. The influence of pure carbohydrate diet on newly emerged honeybees. Annals of the Entomological Society of America. 1937;30:252–62.

[8] Haydak MH. Brood rearing by honeybees confined to a pure carbohydrate diet. Journal of Economic Entomology. 1935;28:657–60.

[9] Crailsheim K. The protein balance of the honey bee worker. Apidologie. 1990;21:417–29.

[10] Brodschneider R, Crailsheim K. Nutrition and health in honey bees. Apidologie. 2010;41(3):278–94. 10.1051/apido/2010012

[11] Alaux C, Ducloz F, Crauser D, Le Conte Y. Diet effects on honeybee immunocompetence. Biology Letters. 2010; 6(4):562–565. 10.1098/rsbl.2009.0986 20089536 PMC2936196

[12] Alaux C, Dantec C, Parrinello H, Le Conte Y. Nutrigenomics in honey bees: digital gene expression analysis of pollen's nutritive effects on healthy and Varroa-parasitized bees. BMC Genomics. 2011;12:496 Epub 2011/10/12. 10.1186/1471-2164-12-496 21985689 PMC3209670

[13] Corby-Harris V, Meador CA, Snyder LA, Schwan MR, Maes P, Jones BM, et al Transcriptional, translational, and physiological signatures of undernourished honey bees (*Apis mellifera*) suggest a role for hormonal factors in hypopharyngeal gland degradation. J Insect Physiol. 2016;85:65–75. Epub 2015/12/15. 10.1016/j.jinsphys.2015.11.016 . 26658137

[14] Corby-Harris V, Jones BM, Walton A, Schwan MR, Anderson KE. Transcriptional markers of sub-optimal nutrition in developing *Apis mellifera* nurse workers. BMC Genomics. 2014;15:134 Epub 2014/02/18. 10.1186/1471-2164-15-134 24529032 PMC3933195

[15] DeGrandi-Hoffman G, Wardell G, Ahumada-Segura F, Rinderer T, Danka R, Pettis J. Comparisons of pollen substitute diets for honey bees: consumption rates by colonies and effects on brood and adult populations. Journal of Apicultural Research. 2008;47:265–70.

[16] Rinderer TE, Rothenbuhler WC, Gochnauer TA. The influence of pollen on the susceptibility of honey-bee larvae to *Bacillus* larvae. Journal of Invertebrate Pathology. 1974;23(3):347–50. Epub 1974/05/01. . 4833177 10.1016/0022-2011(74)90100-1

[17] DeGrandi-Hoffman G, Chen Y, Huang E, Huang MH. The effect of diet on protein concentration, hypopharyngeal gland development and virus load in worker honey bees (*Apis mellifera* L.). J Insect Physiol. 2010;56(9):1184–91. Epub 2010/03/30. 10.1016/j.jinsphys.2010.03.017 . 20346950

[18] DeGrandi-Hoffman G, Chen Y, Rivera R, Carroll M, Chambers M, Hidalgo G, et al Honey bee colonies provided with natural forage have lower pathogen loads and higher overwinter survival than those fed protein supplements. Apidologie. 2016;47(2):186–96. 10.1007/s13592-015-0386-6

[19] DeGrandi-Hoffman G, Chen Y, Huang E, Huang MH. The effect of diet on protein concentration, hypopharyngeal gland development and virus load in worker honey bees (*Apis mellifera* L.). Journal of Insect Physiology. 2010;56(9):1184–91. 10.1016/j.jinsphys.2010.03.017 20346950

[20] Schmehl DR, Teal PE, Frazier JL, Grozinger CM. Genomic analysis of the interaction between pesticide exposure and nutrition in honey bees (*Apis mellifera*). J Insect Physiol. 2014;71:177–90. Epub 2014/12/03. 10.1016/j.jinsphys.2014.10.002 . 25450567

[21] Wahl O, Ulm K. Influence of pollen feeding and physiological condition on pesticide sensitivity of the honey bee *Apis mellifera carnica*. Oecologia. 1983;59(1):106–28. Epub 1983/08/01. 10.1007/BF00388082 . 25024157

[22] Schmidt JO, Thoenes SC, Levin MD. Survival of honey bees, *Apis mellifera* (Hymenoptera: Apidae), fed various pollen sources. Annals of the Entomological Society of America. 1987;80:176–83.

[23] Schmidt LS, Schmidt JO, Rao H, Wang W, Xu L. Feeding preference and survival of young worker honey bees (Hymenoptera: Apidae) fed rape, sesame, and sunflower pollen. Journal of Economic Entomology. 1995;88(6):1591–5. 10.1093/jee/88.6.1591

[24] Pernal SF, Currie RW. Pollen quality of fresh and 1-year-old single pollen diets for worker honey bees (*Apis mellifera* L.). Apidologie. 2000;31(3):387–409.

[25] Winston ML. The Biology of the Honey Bee. Cambridge, Massachusetts: Harvard University Press; 1987 281 p.

[26] Waldbauer G, Friedman S. Self-selection of optimal diets by insects. Annual Review of Entomology. 1991;36(1):43–63.

[27] Behmer ST. Insect herbivore nutrient regulation. Annual Review of Entomology. 2009;54. 10.1146/annurev.ento.54.110807.090537 18764740

[28] Smart MD, Cornman RS, Iwanowicz DD, McDermott-Kubeczko M, Pettis JS, Spivak MS, et al A comparison of honey bee-collected pollen from working agricultural lands using light microscopy and ITS metabarcoding. Environmental Entomology. 2017;46(1):38–49. 10.1093/ee/nvw159 28062536

[29] Richardson RT, Lin C-H, Quijia JO, Riusech NS, Goodell K, Johnson RM. Rank-Based Characterization of pollen assemblages collected by honey bees using a multi-locus metabarcoding approach. Applications in Plant Sciences. 2015;3(11):1500043 10.3732/apps.1500043 26649264 PMC4651628

[30] Cornman RS, Otto CRV, Iwanowicz D, Pettis JS. Taxonomic characterization of honey bee (*Apis mellifera*) pollen foraging based on non-overlapping paired-end sequencing of nuclear ribosomal loci. PloS One. 2015;10(12):e0145365 10.1371/journal.pone.0145365 26700168 PMC4689544

[31] Baum KA, Rubink WL, Coulson RN, Bryant jVM. Pollen selection by feral honey bee (Hymenoptera: Apidae) colonies in a coastal prairie landscape. Environmental Entomology. 2004;33(3):727–39. 10.1603/0046-225x-33.3.727

[32] Leonhardt SD, Blüthgen N. The same, but different: pollen foraging in honeybee and bumblebee colonies. Apidologie. 2012;43(4):449–64. 10.1007/s13592-011-0112-y

[33] Regali A, Rasmont P. Nouvelles méthodes de test pour l'évaluation du régime alimentaire chez des colonies orphelines de Bombus terrestris (L) (Hymenoptera, Apidae). Apidologie. 1995;26(4):273–81.

[34] Ruedenauer FA, Spaethe J, Leonhardt SD. Hungry for quality—individual bumblebees forage flexibly to collect high-quality pollen. Behav Ecol Sociobiol. 2016;70(8):1209–17. 10.1007/s00265-016-2129-8

[35] Nicholls E, de Ibarra NH. Bees associate colour cues with differences in pollen rewards. The Journal of Experimental Biology. 2014;217(15):2783–8. 10.1242/jeb.106120 24855678

[36] Ruedenauer FA, Spaethe J, Leonhardt SD. How to know which food is good for you: bumblebees use taste to discriminate between different concentrations of food differing in nutrient content. The Journal of Experimental Biology. 2015;218(Pt 14):2233–40. Epub 2015/07/24. 10.1242/jeb.118554 . 26202778

[37] Kitaoka TK, Nieh JC. Bumble bee pollen foraging regulation: role of pollen quality, storage levels, and odor. Behav Ecol Sociobiol. 2009;63(4):501–10. 10.1007/s00265-008-0684-3

[38] Cook SM, Awmack CS, Murray DA, Williams IH. Are honey bees' foraging preferences affected by pollen amino acid composition? Ecological Entomology. 2003;28(5):622–7. 10.1046/j.1365-2311.2003.00548.x

[39] Pernal SF, Currie RW. Discrimination and preferences for pollen-based cues by foraging honeybees, *Apis mellifera* L. Animal Behaviour. 2002;63(2):369–90. 10.1006/anbe.2001.1904.

[40] Pernal SF, Currie RW. The influence of pollen quality on foraging behavior in honeybees (*Apis mellifera* L.). Behav Ecol Sociobiol. 2001;51(1):53–68. 10.1007/s002650100412

[41] Waddington KD, Nelson CM, Page RE. Effects of pollen quality and genotype on the dance of foraging honey bees. Animal Behaviour. 1998;56(1):35–9. 10.1006/anbe.1998.0736 9710459

[42] Fowler RE, Rotheray EL, Goulson D. Floral abundance and resource quality influence pollinator choice. Insect Conservation and Diversity. 2016;9(6):481–94. 10.1111/icad.12197

[43] Beekman M, Preece K, Schaerf TM. Dancing for their supper: Do honeybees adjust their recruitment dance in response to the protein content of pollen? Insectes Sociaux. 2016;63(1):117–26. 10.1007/s00040-015-0443-1

[44] Hendriksma HP, Shafir S. Honey bee foragers balance colony nutritional deficiencies. Behav Ecol Sociobiol. 2016;70(4):509–17.

[45] Zarchin S, Dag A, Salomon M, Hendriksma HP, Shafir S. Honey bees dance faster for pollen that complements colony essential fatty acid deficiency. Behav Ecol Sociobiol. 2017;71(12):172 10.1007/s00265-017-2394-1

[46] Crailsheim K, Schneider LHW, Hrassnigg N, Bühlmann G, Brosch U, Gmeinbauer R, et al Pollen consumption and utilization in worker honeybees (*Apis mellifera carnica*): Dependence on individual age and function. Journal of Insect Physiology. 1992;38(6):409–19. 10.1016/0022-1910(92)90117-V.

[47] Moritz B, Crailsheim K. Physiology of protein digestion in the midgut of the honeybee (*Apis mellifera* L.). Journal of Insect Physiology. 1987;33(12):923–31. 10.1016/0022-1910(87)90004-7.

[48] Camazine S. The regulation of pollen foraging by honey bees: how foragers assess the colony's need for pollen. Behav Ecol Sociobiol. 1993;32(4):265–72. 10.1007/bf00166516

[49] Camazine S, Crailsheim K, Hrassnigg N, Robinson G, E, Leonhard B, Kropiunigg H. Protein trophallaxis and the regulation of pollen foraging by honey bees (*Apis mellifera* L.). Apidologie. 1998;29(1–2):113–26.

[50] Sagili RR, Pankiw T. Effects of protein-constrained brood food on honey bee (*Apis mellifera* L.) pollen foraging and colony growth. Behav Ecol Sociobiol. 2007;61(9):1471–8. 10.1007/s00265-007-0379-1

[51] Weidenmüller A, Tautz J. In-hive behavior of pollen foragers (*Apis mellifera*) in honey bee colonies under conditions of high and low pollen need. Ethology. 2002;108(3):205–21. 10.1046/j.1439-0310.2002.00759.x

[52] Dreller C, Tarpy DR. Perception of the pollen need by foragers in a honeybee colony. Animal Behaviour. 2000;59(1):91–6. 10.1006/anbe.1999.1303 10640370

[53] Vaughan DM, Calderone NW. Assessment of pollen stores by foragers in colonies of the honey bee, *Apis mellifera* L. Insectes Sociaux. 2002;49(1):23–7. 10.1007/s00040-002-8273-3

[54] Calderone NW, Johnson BR. The within-nest behaviour of honeybee pollen foragers in colonies with a high or low need for pollen. Animal Behaviour. 2002;63(4):749–58. 10.1006/anbe.2001.1957.

[55] Dreller C, Page RE Jr., Fondrk MK. Regulation of pollen foraging in honeybee colonies: effects of young brood, stored pollen, and empty space. Behav Ecol Sociobiol. 1999;45(3):227–33. 10.1007/s002650050557

[56] Pankiw T, Page RE Jr, Kim Fondrk M. Brood pheromone stimulates pollen foraging in honey bees (*Apis mellifera*). Behav Ecol Sociobiol. 1998;44(3):193–8. 10.1007/s002650050531

[57] Paoli PP, Donley D, Stabler D, Saseendranath A, Nicolson SW, Simpson SJ, et al Nutritional balance of essential amino acids and carbohydrates of the adult worker honeybee depends on age. Amino Acids. 2014;46(6):1449–58. 10.1007/s00726-014-1706-2 24623119 PMC4021167

[58] Lehotay SJ, Maštovská K, Lightfield AR. Use of buffering and other means to improve results of problematic pesticides in a fast and easy method for residue analysis of fruits and vegetables. Journal of AOAC International. 2005;88(2):615–29. 15859090

[59] Little DP, Schwarzbach AE, Adams RP, Hsieh CF. The circumscription and phylogenetic relationships of *Callitropsis* and the newly described genus *Xanthocyparis* (Cupressaceae). American Journal of Botany. 2004;91(11):1872–81. Epub 2004/11/01. 10.3732/ajb.91.11.1872 . 21652334

[60] Wilson EE, Sidhu CS, LeVan KE, Holway DA. Pollen foraging behaviour of solitary Hawaiian bees revealed through molecular pollen analysis. Molecular Ecology. 2010;19(21):4823–9. 10.1111/j.1365-294X.2010.04849.x 20958818

[61] Toth AL, Kantarovich S, Meisel AF, Robinson GE. Nutritional status influences socially regulated foraging ontogeny in honey bees. The Journal of Experimental Biology. 2005;208(Pt 24):4641–9. Epub 2005/12/06. 10.1242/jeb.01956 . 16326945

[62] Vaudo AD, Patch HM, Mortensen DA, Tooker JF, Grozinger CM. Macronutrient ratios in pollen shape bumble bee (*Bombus impatiens*) foraging strategies and floral preferences. Proc Natl Acad Sci U S A. 2016;113(28):E4035–42. Epub 2016/07/01. 10.1073/pnas.1606101113 27357683 PMC4948365

[63] Vaudo AD, Stabler D, Patch HM, Tooker JF, Grozinger CM, Wright GA. Bumble bees regulate their intake of essential protein and lipid pollen macronutrients. The Journal of Experimental Biology. 2016;219(Pt 24):3962–70. Epub 2016/11/02. 10.1242/jeb.140772 . 27742891

[64] Keller A, Danner N, Grimmer G, Ankenbrand M, von der Ohe K, von der Ohe W, et al Evaluating multiplexed next-generation sequencing as a method in palynology for mixed pollen samples. Plant Biology. 2015;17(2):558–66. 10.1111/plb.12251 25270225

[65] Lundin O, Ward KL, Artz DR, Boyle NK, Pitts-Singer TL, Williams NM. Wildflower plantings do not compete with neighboring almond orchards for pollinator visits. Environmental Entomology. 2017;46(3):559–64. Epub 2017/04/06. 10.1093/ee/nvx052 . 28379320

[66] Omar E, Abd-Ella AA, Khodairy MM, Moosbeckhofer R, Crailsheim K, Brodschneider R. Influence of different pollen diets on the development of hypopharyngeal glands and size of acid gland sacs in caged honey bees (*Apis mellifera*). Apidologie. 2017;48(4):425–36. 10.1007/s13592-016-0487-x

[67] Arien Y, Dag A, Zarchin S, Masci T, Shafir S. Omega-3 deficiency impairs honey bee learning. Proc Natl Acad Sci U S A. 2015;112(51):15761–6. Epub 2015/12/09. 10.1073/pnas.1517375112 26644556 PMC4697434

[68] Ricigliano VA, Fitz W, Copeland DC, Mott BM, Maes P, Floyd AS, et al The impact of pollen consumption on honey bee (*Apis mellifera*) digestive physiology and carbohydrate metabolism. Archives of Insect Biochemistry and Physiology. 2017 10.1002/arch.21406 28833462

[69] Corby-Harris V, Maes P, Anderson KE. The bacterial communities associated with honey bee *Apis mellifera* foragers. PloS One. 2014;9(4):e95056 10.1371/journal.pone.0095056 24740297 PMC3989306

[70] Anderson KE, Sheehan TH, Mott BM, Maes P, Snyder L, Schwan MR, et al Microbial ecology of the hive and pollination landscape: bacterial associates from floral nectar, the alimentary tract and stored food of honey bees (*Apis mellifera*). PloS One. 2013;8(12):e83125 Epub 2013/12/21. 10.1371/journal.pone.0083125 24358254 PMC3866269

[71] London-Shafir I, Shafir S, Eisikowitch D. Amygdalin in almond nectar and pollen–facts and possible roles. Plant Systematics and Evolution. 2003;238(1):87–95. 10.1007/s00606-003-0272-y

[72] Hopkins C, Jevans A, Boch R. Occurrence of octadeca-trans-2, cis-9, cis-12-trienoic acid in pollen attractive to the honey bee. Canadian Journal of Biochemistry. 1969;47(4):433–6. 5769090 10.1139/o69-067

[73] Lepage M, Boch R. Pollen lipids attractive to honeybees. Lipids. 1968;3(6):530–4. 10.1007/BF02530897 17805808

