# Supplementary material for: Honey bee (Apis mellifera) nurses do not consume pollens based on their nutritional quality
Source: PLoS One. 2018 Jan 11;13(1):e0191050. doi: 10.1371/journal.pone.0191050 (PMC5764376; doi:10.1371/journal.pone.0191050)
Supplement: S3 File — (PDF) [file pone.0191050.s003.pdf]

DONALD DANFORTH
PLANT SCIENCE CENTER
DISCOVERY | COMMUNITY | IMPACT

Donald Danforth Plant Science Center
Proteomics & Mass Spectrometry Facility

May 2, 2017

Name: Vanessa Corby-Harris
Institution: USDA-ARS
Sample Information:

Date In: Feb 2, 2017
Handler: Jia Li
No. of samples: 3
Requested Procedure(s): GC-FID/FAMEs and GC-MS Sterol Analysis

Results Summary:

For FAMEs analysis the samples were weighed out and dispensed into a screw top glass tubes. 2.5mL of 2.5% H_2SO_4 in methanol, 25 µL of BHT (0.2%) and 30 µL of C17:0 TAG (10mg/ml) was added. The samples were heated at 85˚C for 2 hours. Fatty acid methyl esters were extracted with hexane. Samples were analyzed by GC-FID. The content of the sample oil was calculated base on the internal standard 17:0 TAG. We found that there were two unknown peaks in the corn sample. Oil percent calculations with the two unknown peaks is 6.48%, without the two unknown peaks oil content is 5.85%. Chromatograms and area% reports are attached.

sample name	weight(mg)	oil%	
corn	29	5.85	6.48
rapini	33	4.70	
almond	30	4.87	

For sterol analysis, the samples were weighed out into 2 mL Eppendorf tubes. 100 mg of pollen was extracted with 400 µL hexane. The samples were homogenized in a TissueLyserII at 20 Hz for 10 minutes. Solids were collected by centrifugation and the supernatant was transferred to a GC-vial for analysis. One microliter was injected of each sample. The concentrations of the sterols detected were determined using a standard curve using cholesterol. The concentrations are tabulated below. Chromatograms and spectra are attached. Identifications for some species is ambiguous. For that reason, peak identities are denoted by their retention time in the table below.

		Sample (M)		
		Almond	Corn	Rapini
Retention Time	9.12	0.000254742	0.000103	1.29E-05
	9.21	1.36859E-05	1.38E-05	ND
	9.36	ND	1.69E-05	ND

	9.56	ND	4.13E-05	ND
	9.66	1.04757E-05	ND	ND
	9.77	1.41852E-05	4.28E-05	1.49E-05
	11.30	4.68087E-05	1.25E-05	1.37E-05

Publication acknowledgement: If any results from this method are being used for publication or grants, it is greatly appreciated to acknowledge the Proteomics & Mass Spectrometry Facility at the Danforth Plant Science Center for its contribution.

Thank you for your patronage! Please contact us if you have any questions regarding your results.

Donald Danforth Plant Science Center
Proteomics & Mass Spectrometry Facility
975 N Warson Rd.
St. Louis, MO 63132
Fax: 314.587.1324
http://www.danforthcenter.org/scientists-research/core-technologies/proteomics-mass-spectrometry

Brad Evans, Ph.D.	Director	bevans@danforthcenter.org	314-587-1464
Shin-Cheng Tzeng, Ph.D.	Staff Scientist	stzeng@danforthcenter.org	314-587-1415
Jonathan Mattingly	Research Associate	jmattingly@danforthcenter.org	314-587-1461
Jia Li	Senior Lab Tech	jli@danforthcenter.org	314-587-1623

Area % Report

Data File: C:\ChromQuest\Data\JL\2017\03-2017\20170309b\004.dat
Method: C:\ChromQuest\Enterprise\Projects\Default\Method\Jia\02282017b.met almond
Acquired: 3/9/2017 8:32:25 PM
Printed: 3/10/2017 10:07:42 AM

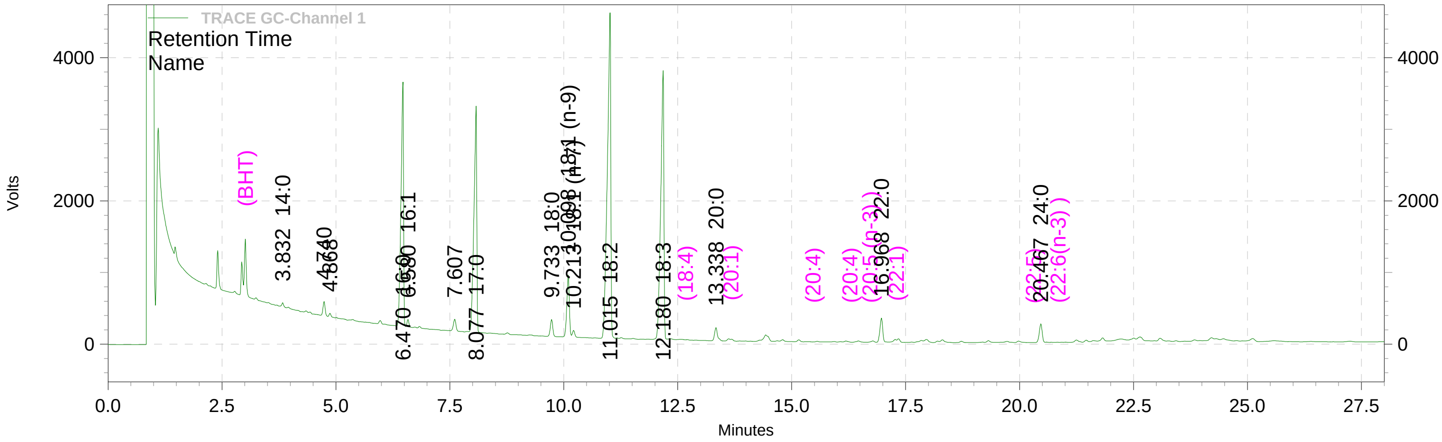

TRACE GC-Channel 1 Results (System (3/10/2017 10:04:53 AM) (Reprocessed))

Retention Time	Area	Area %	Height	Height %	Name
3.832	836352	0.12	389297	0.25	**14:0**
4.740	5306592	0.76	1740544	1.11	
4.868	1238880	0.18	447532	0.28	
6.470	117244335	16.80	30997728	19.71	**16:0**
6.580	2720223	0.39	949595	0.60	**16:1**
7.607	4941836	0.71	1393025	0.89	
8.077	122273342	17.52	28675603	18.23	**17:0**
9.733	7479159	1.07	2082736	1.32	**18:0**
10.098	29623017	4.24	7781724	4.95	**18:1 (n-9)**
10.213	2871833	0.41	834512	0.53	**18:1 (n-7)**
11.015	215964486	30.94	41257054	26.23	**18:2**
12.180	160829463	23.04	34027311	21.63	**18:3**
13.338	5368495	0.77	1484931	0.94	**20:0**
16.968	11882372	1.70	2986631	1.90	**22:0**
20.467	9450222	1.35	2258718	1.44	**24:0**
Totals	698030607	100.00	157306941	100.00	

Area % Report

Data File: C:\ChromQuest\Data\JL\2017\03-2017\20170309b\002.dat
Method: C:\ChromQuest\Enterprise\Projects\Default\Method\Jia\02282017b.met corn
Acquired: 3/9/2017 7:29:20 PM
Printed: 3/10/2017 9:42:23 AM

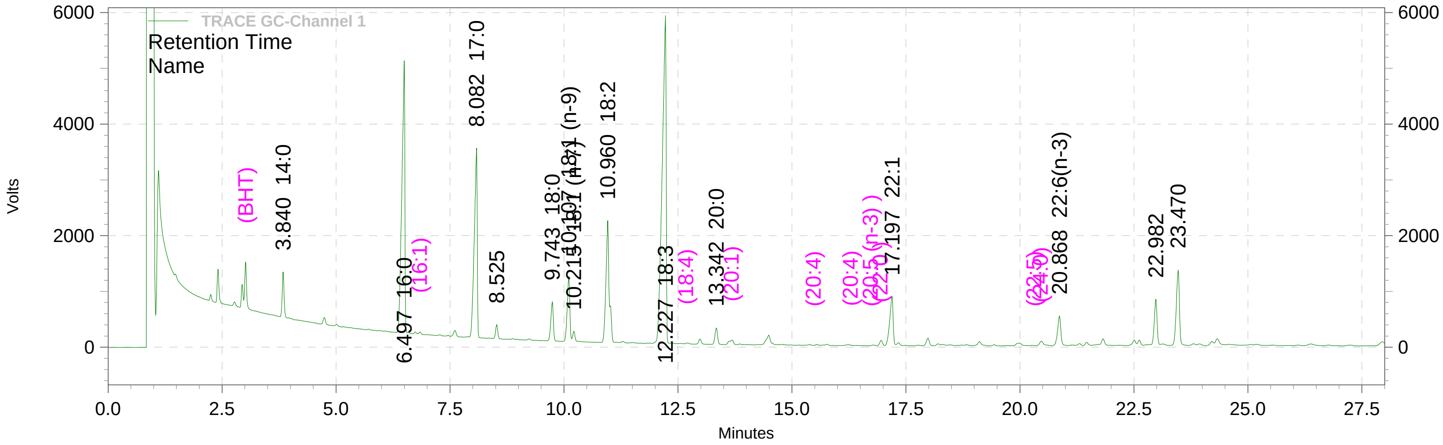

TRACE GC-Channel 1 Results (System (3/10/2017 9:39:42 AM) (Reprocessed))

Retention Time	Area	Area %	Height	Height %	Name
3.840	19733504	2.02	7238202	3.47	**14:0**
6.497	196129248	20.04	44247058	21.21	**16:0**
8.082	134701349	13.77	30769648	14.75	**17:0**
8.525	5724680	0.58	2020992	0.97	
9.743	22223228	2.27	6165939	2.96	**18:0**
10.107	38773033	3.96	10286093	4.93	**18:1 (n-9)**
10.215	4794679	0.49	1504046	0.72	**18:1 (n-7)**
10.960	96401180	9.85	19637202	9.42	**18:2**
12.227	315785753	32.27	53205534	25.51	**18:3**
13.342	8091734	0.83	2374994	1.14	**20:0**
17.197	35911824	3.67	7617497	3.65	**22:1**
20.868	17957781	1.84	4336248	2.08	**22:6(n-3)**
22.982	28317198	2.89	7172752	3.44	
23.470	54033135	5.52	11991585	5.75	
Totals	978578326	100.00	208567790	100.00	

Area % Report

Data File: C:\ChromQuest\Data\JL\2017\03-2017\20170309b\003.dat
Method: C:\ChromQuest\Enterprise\Projects\Default\Method\Jia\02282017b.met rapini
Acquired: 3/9/2017 8:00:51 PM
Printed: 3/10/2017 10:03:27 AM

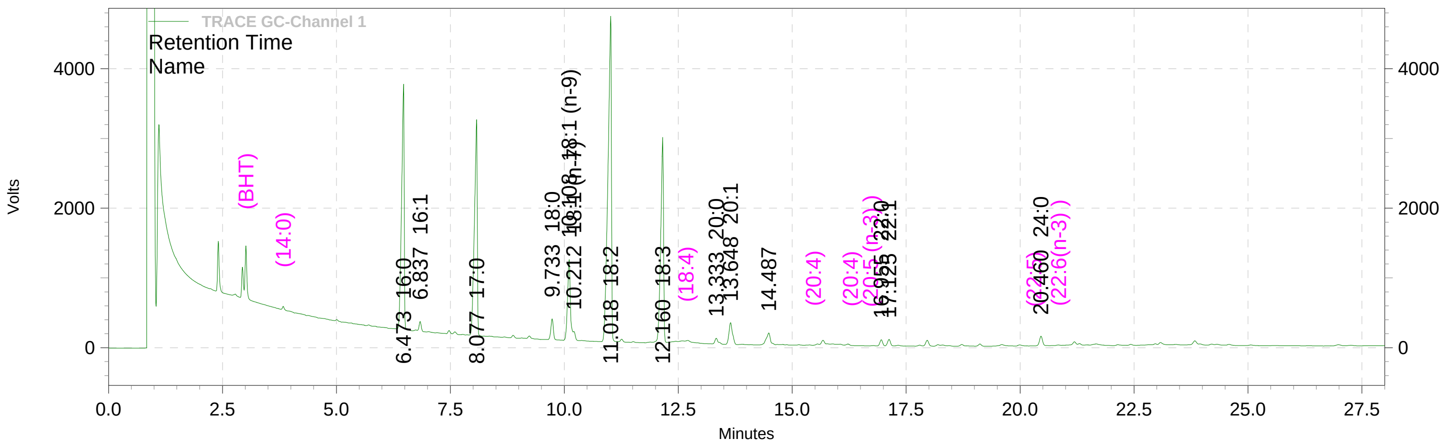

TRACE GC-Channel 1 Results (System (3/10/2017 9:54:31 AM) (Reprocessed))

Retention Time	Area	Area %	Height	Height %	Name
6.473	121983857	18.30	31953104	21.19	**16:0**
6.837	3272856	0.49	1149508	0.76	**16:1**
8.077	120243363	18.04	28108905	18.64	**17:0**
9.733	9374386	1.41	2622993	1.74	**18:0**
10.108	44649348	6.70	10415739	6.91	**18:1 (n-9)**
10.212	3309252	0.50	1110460	0.74	**18:1 (n-7)**
11.018	223580025	33.54	42351553	28.08	**18:2**
12.160	113040955	16.96	26604341	17.64	**18:3**
13.333	1438559	0.22	506620	0.34	**20:0**
13.648	13721722	2.06	2707829	1.80	**20:1**
14.487	2248220	0.34	785208	0.52	
16.955	1898593	0.28	582839	0.39	**22:0**
17.125	3671857	0.55	848266	0.56	**22:1**
20.460	4205080	0.63	1079133	0.72	**24:0**

Totals	666638073	100.00	150826498	100.00	
